# The pathophysiology of bile acid diarrhoea: differences in the colonic microbiome, metabolome and bile acids

**DOI:** 10.1038/s41598-020-77374-7

**Published:** 2020-11-24

**Authors:** Nidhi M. Sagar, Henri Duboc, Gemma L. Kay, Mohammad T. Alam, Alfian N. Wicaksono, James A. Covington, Christopher Quince, Margarita Kokkorou, Vaios Svolos, Lola J. Palmieri, Konstantinos Gerasimidis, Julian R. F. Walters, Ramesh P. Arasaradnam

**Affiliations:** 1 Warwick Medical School, University of Warwick, Coventry, CV4 7AL UK; 2 Louis Mourier Hospital, DHU Unity APHP, and Inserm UMR 1149, Team BADO, UFR de Médecine Paris Diderot, Sorbonne Paris Cité, Paris, France; 3 School of Engineering, University of Warwick, Coventry, CV4 7AL UK; 4 School of Medicine, University of Glasgow, Glasgow, G12 8QQ UK; 5 Division of Digestive Diseases, Imperial College London, and Imperial College Healthcare, London, W12 0HS UK; 6 Division of Gastroenterology, University Hospitals Coventry and Warwickshire, Coventry, CV2 2DX UK; 7 Biological Sciences, Coventry University, Coventry, CV1 5FB UK; 8 Quadram Institute Bioscience, Norwich Research Park, Norwich, NR4 7UQ UK

**Keywords:** Gastrointestinal diseases, Gastroenterology, Gastrointestinal system, Microbiota, Small intestine

## Abstract

Bile acid diarrhoea (BAD) is a common disorder resulting from increased loss of bile acids (BAs), overlapping irritable bowel syndrome with diarrhoea (IBS-D). The gut microbiota metabolises primary BAs to secondary BAs, with differing impacts on metabolism and homeostasis. The aim of this study was to profile the microbiome, metabolic products and bile acids in BAD. Patients with BAD diagnosed by SeHCAT testing, were compared with other IBS-D patients, and healthy controls. Faecal 16S ribosomal RNA gene analysis was undertaken. Faecal short chain fatty acid (SCFA) and urinary volatile organic compounds (VOCs) were measured. BAs were quantified in serum and faeces. Faecal bacterial diversity was significantly reduced in patients with BAD. Several taxa were enriched compared to IBS-D. SCFA amounts differed in BAD, controls and IBS-D, with significantly more propionate in BAD. Separation of VOC profiles was evident, but the greatest discrimination was between IBS-D and controls. Unconjugated and primary BA in serum and faeces were significantly higher in BAD. The faecal percentage primary BA was inversely related to SeHCAT. BAD produces dysbiosis, with metabolite differences, including VOC, SCFA and primary BAs when compared to IBS-D. These findings provide new mechanistic insights into the pathophysiology of BAD.

## Introduction

Bile acid diarrhoea (BAD) is a commonly missed cause of chronic diarrhoea and has been demonstrated in excess of a quarter of patients who were previously diagnosed with IBS-D^1,2^. Functional bowel disorders such as IBS form the largest group of patients seen in a general gastroenterology clinic, but clinicians often neglect the opportunity to diagnose BAD. The established gold standard diagnostic tests for BAD, faecal bile acid measurements, ^75^SeHCAT scan or serum 7α-hydroxy-4-cholesten-3-one (C4), are limited in their availability^3^.

BAD may be due to malabsorption or overproduction of BAs. The ileal hormone fibroblast growth factor 19 (FGF19) regulates hepatic BA synthesis and is low in BAD^4^. Bile acids undergo an enterohepatic circulation. Less than 5% of the glycine- and taurine-conjugated primary BAs, cholic acid (CA) and chenodeoxycholic acid (CDCA), escape active absorption in the terminal ileum^5^. Unabsorbed primary BAs can undergo biotransformation by the microbiota in the colon, to form the secondary BAs, deoxycholic acid (DCA), lithocholic acid (LCA) and ursodeoxycholic acid (UDCA). These are partially absorbed passively in the colon, or excreted in the faeces^5^. Biotransformation enzymatic reactions comprise, first, deconjugation, via bile salt hydrolase (BSH), which catalyses the hydrolysis of glycine or taurine from the C24 N-acyl amide bond of conjugated BAs^6^. Epimerization, oxidation, dehydroxylation and hydroxylation by hydroxysteroid dehydrogenase (HSDHs) enzymes can then occur. 7α-dehydroxylation of primary BAs, forming secondary BAs, is the most quantitatively important and complex microbial bile salt transformation^5–8^.

BA act as signalling molecules through activation of receptors: farnesoid X receptor (FXR) is most potently stimulated by CDCA, and the secondary BAs stimulate TGR5 (also known as G-protein-coupled BA receptor 1, GPBAR-1). BA regulate intestinal homeostasis by inhibiting inflammation, preventing pathogen invasion and maintaining cell integrity, and furthermore stimulate production of hormones including FGF19, GLP-1 and PYY^5^.

Increased colonic exposure to BAs influences stool volume, colonic transit time and bowel habit^5^. The dihydroxy-BAs, DCA and CDCA, stimulate water secretion^9^ and CDCA accelerates colonic transit, increases stool frequency and decreases stool consistency. Increased faecal primary BAs have been demonstrated in IBS-D and have been suggested as a diagnostic biomarker for BAD^10–13^.

Data on the faecal or colonic microbial composition in IBS subjects are inconsistent and sometimes contradictory^14–18^. Only recently have studies including IBS-D patients taken into account the possibility that some will have BAD^19,20^. Dysbiosis occurs when imbalances in gut bacteria precipitate disease and has been linked to the changes in metabolism of BAs in the gut^21^. Evidence suggests that dysbiosis may be secondary to a reduction in bacteria bearing BSH activity^8,22^.

Volatile organic compounds (VOCs) are the resultant gas by-products of colonic fermentation by gut bacteria, which derive energy through oxidation of organic compounds. Changes in VOC, measured in breath, faeces or urine, indirectly reflect changes of the metabolome. A reduction in the number of faecal VOCs in certain diarrhoeal conditions has been observed, suggesting reduced overall biodiversity of the gut flora and decreased synthesis of compounds, perhaps due to more rapid intestinal transit^23^. We expect patients with IBS-D and BAD to exhibit altered VOC profiles compared to healthy controls (HCs)^24^, reflecting underlying dysbiosis with altered metabolic output. Furthermore, rapid intestinal transit could reduce the time for bacteria to metabolise and absorb nutrients and their products, in particular, the short-chain fatty acids (SCFAs). The three primary SCFAs produced by the microbiome are acetate, propionate and butyrate and are integral to health, including providing 5–10% of human basal energy requirements^25,26^.

The objective of this study was to define mechanisms associated with the pathophysiology of BAD, through characterisation of the colonic microbiome, SCFA, VOC metabolites, and quantification of serum and faecal BAs.

## Results

In this exploratory mechanistic study, 156 subjects participated, including 62 with BAD, 55 with IBS-D and 39 healthy controls (HC). Different subsets of patients were involved in the various parts of the study; the demographic and clinical characteristics are outlined in Supplemental Table 1.

**Table 1:** Short chain fatty acids in healthy controls, IBS-D and bile acid diarrhoea subjects.

	Healthy controls		IBS-D		BAD	
	Median	IQR	Median	IQR	Median	IQR
% Faecal Water	69.3	(63.0–72.9)	71.7	(67.6–82.8)	75.6	(64.5–81.2)
Total SCFA	522.9	(418.7–703.5)	465.1	(308.7–944.7)	545.2	(417.8–1075.9)
Acetate	329.9	(252.7–451.5)	286.0	(186.7–610.0)	353.7	(265.7–708.0)
Propionate	78.1	(49.0–103.6)	81.5	(41.9–129.3)	128.4	(51.3–207.7)
Butyrate	73.6	(46.5–100.7)	74.7	(37.7–138.3)	65.4	(36.7–155.2)
Isobutyrate	8.1	(6.0–10.8)	9.3	(7.0–11.9)	7.0	(3.7–11.3)
Valeric	10.9	(7.6–16.7)	11.7	(7.7–15.8)	6.9	(2.4–16.8)
Isovaleric	7.5	(5.6–10.6)	9.7	(6.0–11.4)	8.1	(6.3–12.5)
Caproic	4.7	(1.0–7.8)	2.9	(1.0–9.8)	1.2	(1.0–2.1)
Isocaproic	0.17	(0.13–0.26)	0.22	(0.15–0.48)	0.32	(0.25–0.58)
Heptanoic	0.42	(0.06–1.04)	0.22	(0.06–1.19)	0.09	(0.05–0.25)
Octanoic	0.14	(0.00–0.32)	0.09	(0.0–0.29)	0.06	(0.0–0.26)
% Acetate	65.1	(61.5–67.9)	62.5	(58.4–67.5)	60.1	(53.0–72.5)
% Propionate	14.8	(13.4–16.2)	14.2	(11.2–18.2)	16.8	(13.5–25.3)
% Butyrate	13.8	(11.5–15.1)	13.8	(11.9–16.3)	10.6	(7.7–17.3)
% Isobutyrate	1.6	(1.3–2.0)	2.1	(0.8–3.1)	1.1	(0.5–1.7)
% Valeric	2.2	(1.8–2.5)	2.2	(1.4–2.7)	1.3	(0.3–2.3)
% Isovaleric	1.6	(1.2–2.0)	2.1	(0.6–3.2)	1.7	(0.9–2.3)
% Caproic	0.70	(0.31–1.45)	0.84	(0.36–1.56)	0.22	(0.12–0.43)
% Isocaproic	0.03	(0.02–0.06)	0.04	(0.03–0.09)	0.05	(0.04–0.08)
% Heptanoic	0.07	(0.01–0.21)	0.03	(0.01–0.21)	0.02	(0.01–0.04)
% Octanoic	0.03	(0.01–0.06)	0.02	(0.00–0.06)	0.01	(0.0–0–0.04)

	Ratios			*P* value		
	IBS/HC	BAD/HC	BAD/IBS	Overall	BAD/HC	BAD/IBS
% Faecal Water	1.04	1.09	1.05	**0.02**	**0.01**	0.81
Total SCFA	0.89	1.04	1.17	0.32	0.16	0.21
Acetate	0.87	1.07	1.24	0.31	0.24	0.17
Propionate	1.04	1.64	1.58	0.11	**0.04**	0.11
Butyrate	1.01	0.89	0.88	0.99	0.98	0.89
Isobutyrate	1.15	0.86	0.75	0.40	0.41	0.28
Valeric	1.07	0.63	0.59	0.41	0.24	0.26
Isovaleric	1.29	1.08	0.84	0.61	0.43	0.98
Caproic	0.61	0.26	0.43	0.11	**0.04**	0.14
Isocaproic	1.31	1.87	1.43	**0.01**	**0.003**	0.09
Heptanoic	0.52	0.22	0.43	0.14	**0.05**	0.15
Octanoic	0.62	0.42	0.68	0.91	0.78	0.64
% Acetate	0.96	0.92	0.96	0.39	0.25	0.79
% Propionate	0.96	1.13	1.18	0.19	0.12	0.13
% Butyrate	1.00	0.77	0.77	0.47	0.27	0.30
% Isobutyrate	1.29	0.65	0.50	**0.00**	**0.04**	**0.06**
% Valeric	1.01	0.58	0.58	**0.04**	**0.02**	**0.04**
% Isovaleric	1.32	1.08	0.82	0.70	0.79	0.42
% Caproic	1.19	0.31	0.26	**0.007**	**0.001**	**0.003**
% Isocaproic	1.29	1.41	1.09	0.11	**0.05**	0.57
% Heptanoic	0.45	0.27	0.60	0.09	**0.03**	0.10
% Octanoic	0.77	0.39	0.51	0.85	0.69	0.55

Data are expressed as medians and interquartile ranges (IQR). Total SCFA and individual SCFA are expressed as µmol g^−1^ dry weight.

Data from healthy controls (n = 26), IBS-D (n = 20) and BAD (n = 20) groups.

Statistical comparisons were by Kruskal Wallis tests between all three groups and Mann Witney U-test between two groups. *P* values < 0.05 are shown in bold.

### Microbiome

**Figure 1:**
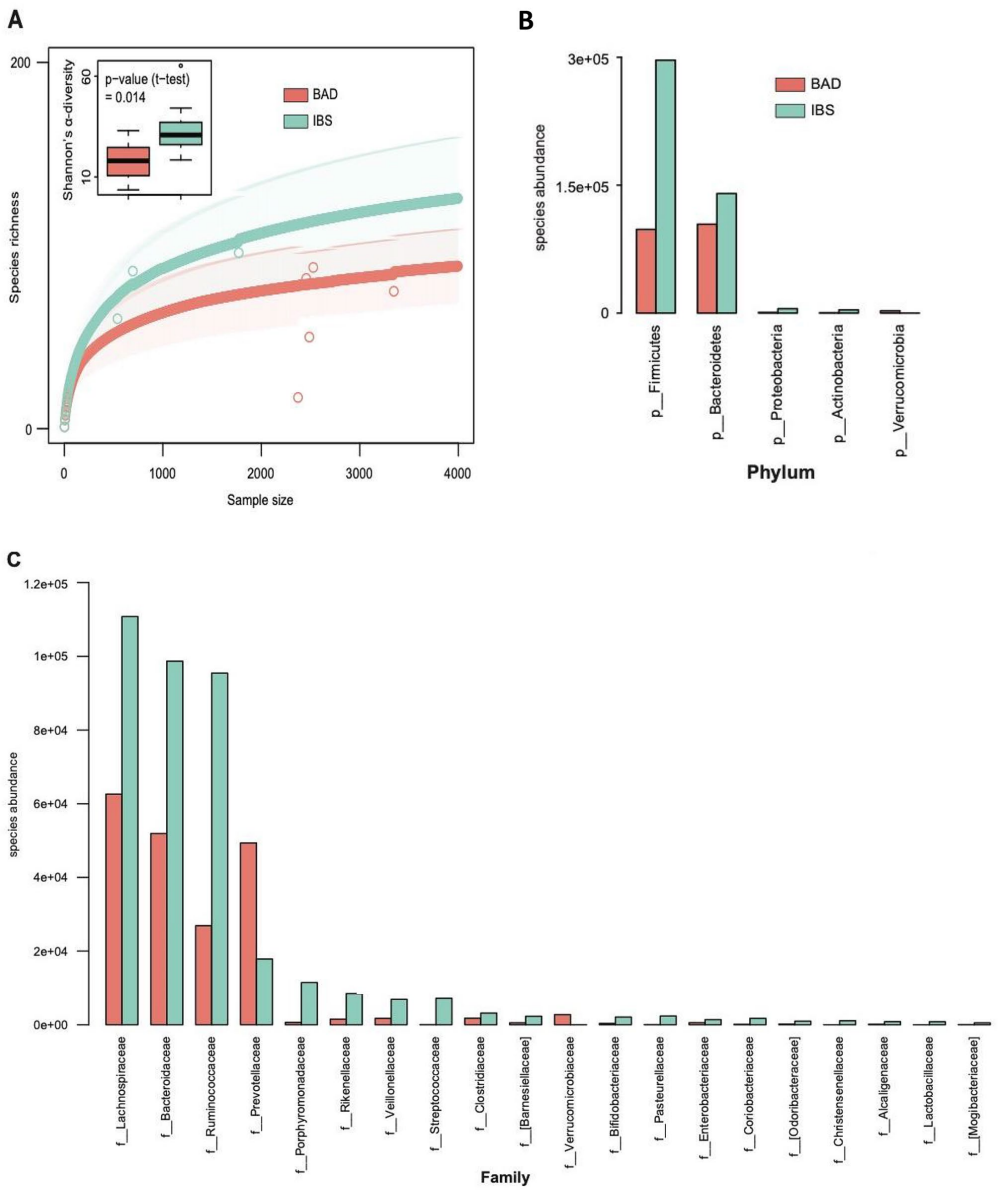
Taxonomic analysis of data from16S rRNA gene sequencing of faecal microbiota in patients with BAD and IBS-D. (**A**) Rarefaction curve and Shannon’s diversity box plot (inset) analysing the diversity of OTUs in BAD compared to IBS. (**B**) Phylum level differences in abundance of assigned species between BAD and IBS-D. (**C**) Family level differences in abundance of assigned species between BAD and IBS.

668 operational taxonomic units (OTUs) were identified from the faecal microbiota in patients from the BAD and IBS-D groups. There was statistically significant difference in the alpha-diversity of OTUs with reduced bacterial diversity in BAD compared to IBS-D. This is demonstrated in the rarefaction curve (*p* = 0.01), and Shannon’s diversity box plot (*p* = 0.014) shown in Fig. 1A.

Taxa identified from these OTUs showed, at the phylum level, that Firmicutes were most abundant in IBS-D, but in BAD they were more reduced compared to the reduction of Bacteroidetes (Fig. 1B). Most families, including Lachnospiraceae, Ruminococcaceae, and Bacteroidaceae were lower in abundance in BAD, but there were increases in Prevotellaceae and Verrcomicrobiaceae (Fig. 1C) Differences in the abundances of many OTUs were found, but the statistical significances of these were not robust when *p* values were adjusted for multiple comparisons. The 10 OTUs that were most significantly enriched in BAD (all uncorrected *p* < 0.01) were two unidentified members of the Lachnospiraceae family {OTU_136 and 268}, another from the Ruminococcaceae family {OTU_356}, a member of the *Ruminococcus* genus {OTU_519}, *Bifidobacterium longum* {OTU_283}, *Prevotella copri* {OTU_17 and OTU_127}, *Akkermansia muciniphila* {OTU_319} and two members of the *Bacteroides* genus {OTU_72 and OTU_553} (Supplemental Table 2).

**Table 2:** Correlation coefficients of fecal water, short chain fatty acids and SeHCAT.

	% Fecal water		SeHCAT %	
	Rs	*p*	Rs	*p*
SeHCAT	0.08	0.33	–	–
Total SCFA	0.60	**0.0001**	− 0.07	0.35
Acetate	0.67	**0.0001**	− 0.09	0.30
Propionate	0.57	**0.0001**	− 0.11	0.25
Butyrate	0.42	**0.004**	0.07	0.35
Isobutyrate	− 0.08	0.32	0.15	0.18
Valeric	0.01	0.48	0.30	**0.03**
Isovaleric	− 0.31	**0.03**	− 0.05	0.39
Caproic	− 0.11	0.26	0.28	**0.05**
Isocaproic	0.20	0.12	− 0.24	0.08
Heptanoic	− 0.06	0.35	0.27	0.05
Octanoic	− 0.09	0.29	0.04	0.41
% Acetate	− 0.03	0.42	0.10	0.28
% Propionate	0.18	0.14	− 0.19	0.13
% Butyrate	− 0.02	0.46	0.20	0.12
% Isobutyrate	− 0.54	**0.0003**	0.24	0.07
% Valeric	− 0.56	**0.0001**	0.33	**0.02**
% Isovaleric	− 0.64	**0.0001**	0.06	0.36
% Caproic	− 0.36	**0.01**	0.39	**0.01**
% Isocaproic	− 0.41	**0.005**	− 0.23	0.09
% Heptanoic	− 0.24	0.14	0.21	0.10
% Octanoic	− 0.18	0.14	0.06	0.35

SCFA amounts were measured as µmol∙g^−1^ of dry stool. SeHCAT % retention at 7d.

N = 38 patients in the combined IBS-D and BAD groups with SeHCAT results.

Rs = Spearman rank correlation coefficients.

*P* values < 0.05 are shown in bold.

### Faecal SCFAs

Faecal water content differed between the three groups (Kruskal–Wallis, *p* = 0.02). The median water content was higher in the BAD group (75.6%) and IBS-D (71.7%) compared to the HC (69.3%; *p* = 0.01 and 0.04 respectively). The total amount of SCFA varied between individuals, particularly in the IBS-D group, but was not significantly different between the three groups (Table 1). In each group, acetate was the most prevalent SCFA. Comparing all the groups, there were significant differences (Kruskal–Wallis, *p* < 0.05) in the concentration of isocaproic, and in the proportions of isobutyric, valeric and caproic.

Direct comparison of the BAD and HC groups showed significantly greater concentrations of propionate (*p* = 0.04) and isocaproic (*p* < 0.01) in the BAD cohort. The increase in proportion of propionate was less significant (*p* = 0.12). The proportion of isobutyrate was significantly lower (*p* = 0.04). Concentrations of caproic and heptanoic were significantly lower, as were their proportions and that of valeric. The proportion of isocaproic was higher. Comparison of the BAD and IBS-D groups gave broadly similar differences to those found in the comparison of BAD and HC, although the significance was usually weaker. There were no significant differences between the IBS-D and HC cohorts.

The specific SCFA associations with the total amount of SCFA in each group showed differences again in the associations of minor SCFA (caproic, heptanoic, and octanoic) with total SCFA in the separate groups, with stronger negative correlations in the BAD group (Supplemental Table 3). In the combined IBS-D and BAD groups, there were strong associations between individual SCFA values and the percentage of faecal water, but not with SeHCAT values (Table 2).

**Table 3:** Predictive values of VOC analysis for different groups.

Comparison	AUC	Sensitivity	Specificity	PPV	NPV	*P* value
BAD versus HC	70% (49–91%)	69% (39–91%)	69% (39–91%)	69%	69%	**0.042**
BAD versus IBS	67% (49–85%)	85% (55–98%)	46% (27–67%)	44%	86%	**0.041**
IBS versus HC	95% (89–100%)	89% (70–98%)	92% (64–100%)	96%	80%	**< 0.001**

AUC, area under the curve; PPV, positive predictive value; NPV negative predictive value; Numbers in brackets are 95% confidence intervals.

### Urinary VOC profiles

To analyse urinary VOCs, five different classification algorithms were assessed. The Support Vector Machine algorithm produced the best results to predict the probability of a sample being from one of the three groups of HC, IBS-D or BAD. Predictive values comparing pairs of groups are shown in Table 3. There were subtle but significant differences between BAD and HC (sensitivity and specificity 69%, *p* = 0.042) and between BAD and IBS-D (sensitivity 85% specificity 46%, *p* = 0.041). However, there was a clearly significant separation of VOC profiles between the IBS-D and HC groups (sensitivity 88%, specificity 92%, *p* < 0.001).

### BA profiles: serum

Comparing the BAD and IBS-D patients with known SeHCAT tests, there was considerable individual variability, and total serum BA were similar in both groups: medians 2.08 (IQR 1.13–4.15) and 1.94 μmol L^−1^ (1.33–3.11). Total secondary BA were lower in the BAD group at 0.56 (0–1.34) versus 1.04 μmol L^−1^ (0.49–1.51), *p* = 0.22, and were very low or absent in three subjects with severe BAD. The amounts of DCA, LCA, tauro- and sulfo-conjugates were reduced to 40–70% with *p* values between 0.13 and 0.28.

Individual serum BA when expressed as a proportion of the total showed broadly similar findings. The median percentage of LCA was lower in BAD, at 2.9% vs. 9.8% in IBS-D (*p* = 0.09). The median percentages for total glyco-, tauro- and sulfo-conjugates were all reduced in BAD to around half of the values in IBS-D (*p* = 0.09, 0.03, 0.11 respectively). The proportions of GCDCA and UDCA were not increased.

**Figure 2:**
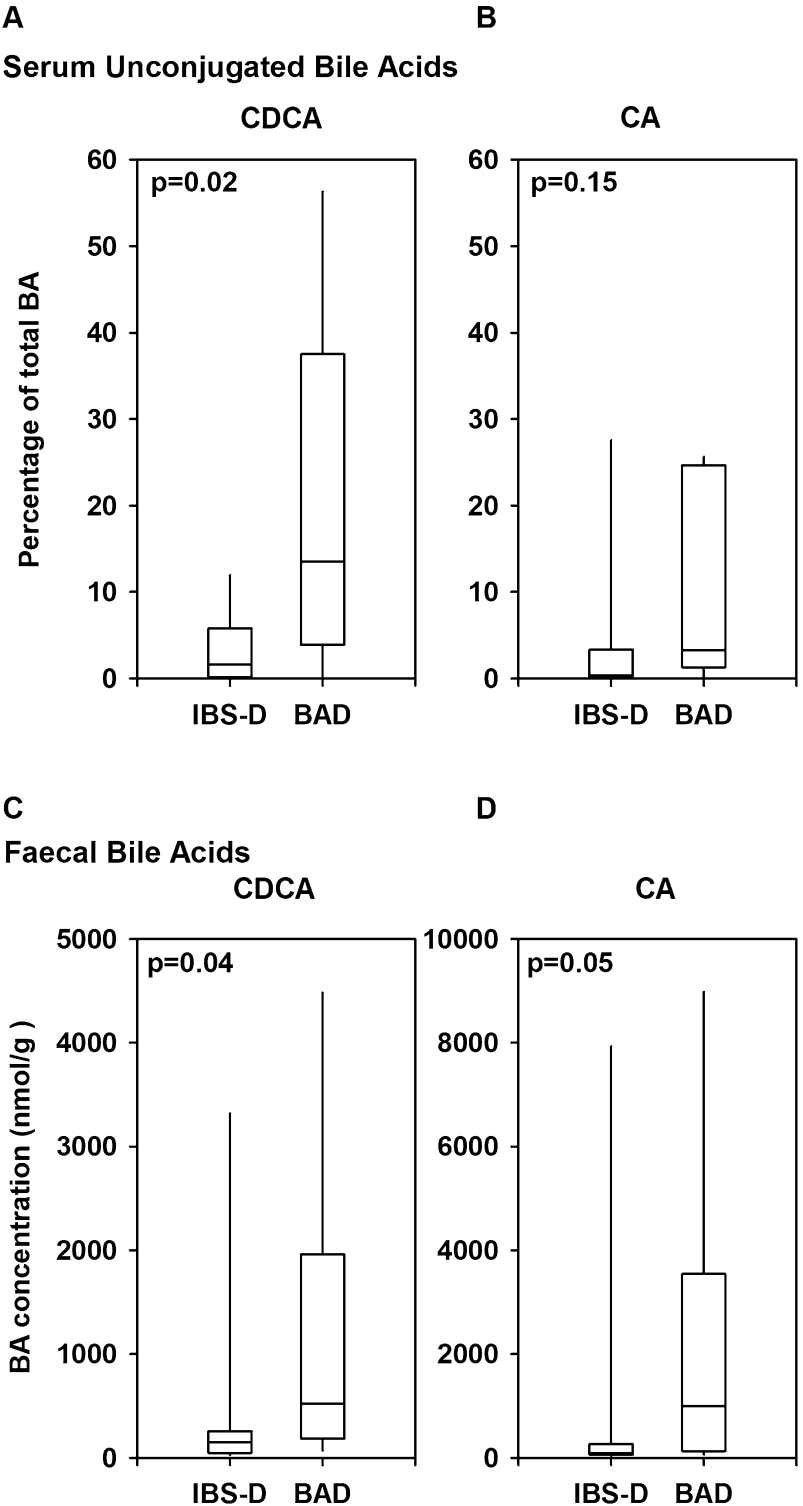
Specific primary bile acid differences in serum and faeces. The percentage of total serum bile acids is shown, in (**A**) unconjugated chenodeoxycholic acid (CDCA); (**B**) unconjugated cholic acid (CA). Concentration in faeces is shown in (**C**) CDCA; (**D**) CA. Each figure shows medians, 25th and 75th centiles, and ranges for IBS-D and BAD patients. *P*-values are for comparisons of the two groups by Mann–Whitney testing.

Along with the reduction in conjugated BA, there were increases in the proportion of unconjugated (free) BAs, to a median of 55.6% (25.6–72.4) in BAD vs. 21.5% (7.0–55.5) in IBS-D (*p* = 0.08). The unconjugated primary BAs were specifically increased (Fig. 2A,B); the median percentage of serum unconjugated CDCA was 13.5% (3.9–37.5) in BAD vs. 1.6% (0.1–5.8) in IBS-D (*p* = 0.02), with a similar ninefold difference found for unconjugated cholic acid (*p* = 0.15), but not for DCA (*p* = 0.96).

### Faecal BA

The individual faecal BA composition on a single sample showed considerable variation, but several differences were apparent between the two groups. The median total faecal BA in the BAD group was about twofold higher than in the IBS-D group, at 9.17 (IQR 7.79–14.12) vs. 4.72 µmol g^−1^ (2.26–6.17), *p* = 0.01, Table 4. The median total primary BA was sixfold higher, with both cholic acid 11.6-fold and CDCA 3.5-fold higher, each *p* < 0.05, (Fig. 2C,D). Medians for the various secondary BAs were also higher, but were less marked, not reaching statistical significance. Other notable findings were higher amounts of total UDCA (13.5-fold, *p* = 0.06), sulfated BA (3.4-fold, *p* = 0.03), and the sum of all unconjugated BA excluding UDCA (1.9-fold, *p* = 0.01).

**Table 4:** Faecal bile acids in patients with IBS-D or bile acid diarrhoea.

BA concentration (nmol∙g^−1^)	IBS-D		BAD		*P* values
	Median	IQR	Median	IQR	
Total faecal BA	4716	(2259–6168)	9170	(7791–14,118)	**0.01**
Primary BA	246	(107–476)	1502	(299–7222)	**0.03**
CA	86	(61–266)	995	(129–3544)	**0.05**
CDCA	149	(48–256)	523	(184–1961)	**0.04**
Secondary BA	4412	(1979–5725)	7130	(3200–9626)	0.14
DCA	3661	(1234–4670)	5295	(2734–7469)	0.14
LCA	766	(675–1240)	1447	(466–2338)	0.25
UDCA	17	(9–52)	232	(27–702)	**0.06**
Glycoconjugates	67	(29–168)	79	(61–107)	0.81
Tauroconjugates	26	(17–59)	25	(12–95)	0.93
Sulfoconjugates	30	(13–91)	101	(73–553)	**0.03**
Unconjugated BA–Urso	4484	(2132–5793)	8465	(7145–11,723)	**0.01**
Ratio primary/secondary	0.08	(0.03–0.13)	0.19	(0.04–5.26)	0.37

BA were measured in a single stool sample from patients with IBS-D with (SeHCAT > 15%; n = 9), or BAD (SeHCAT < 15%; n = 10).

Comparisons were made by Mann–Whitney U-tests.

*P* values < 0.05 are shown in bold.

The percentage proportion of primary BA (%PBA) in the total was double (median 14.3% BAD vs. 7.1% IBS-D), with both cholic acid and CDCA increased (Supplemental Table 4). There were corresponding reductions in total secondary BA, DCA and LCA. The percentage of UDCA was higher, but none of these percentage changes reached significance.

**Figure 3:**
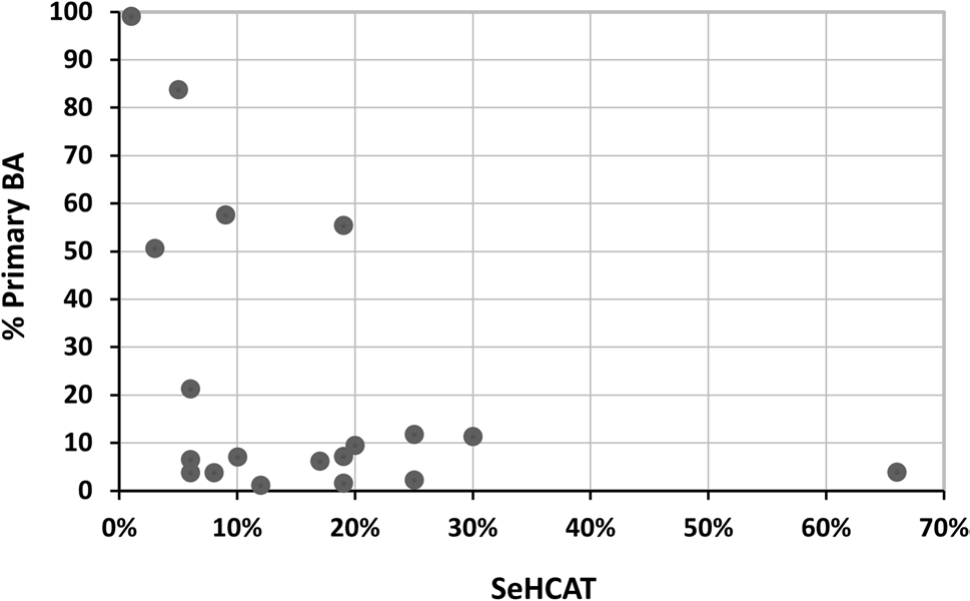
The relationship between percentage of faecal total primary BAs and SeHCAT retention. Values for the percentage of total primary BA (cholic and chenodeoxycholic acids) in faeces and SeHCAT retention are shown for 10 patients with SeHCAT < 15% (BAD) and 9 patients with SeHCAT > 15% (IBS-D).

The data were analysed to see whether BA measured in a single stool sample correlated with the SeHCAT result. In the combined group of BAD and IBS-D patients, SeHCAT retention was inversely related to total faecal BA (Rs =  − 0.53, *p* < 0.01). The individual values for %PBA and SeHCAT, shown in Fig. 3, were variable. The relationship between %PBA and total BA was weaker, not reaching significance (Rs = 0.22, *p* = 0.19). The percentages of the individual primary BA acids (CA or CDCA) were also negatively associated with SeHCAT, whereas the secondary BA, except UDCA, were positively related (Table 5).

**Table 5:** Correlation of SeHCAT and faecal BA percentages.

	Correlation (Rs)	*p *value
Total BA	− 0.53	**0.01**
**Percentages**		
Cholate	− 0.40	**0.05**
Chenodeoxycholate	− 0.41	**0.04**
Total primary BA	− 0.37	**0.06**
Deoxycholate	0.40	**0.05**
Lithocholate	0.59	**0.004**
Ursodeoxycholate	− 0.20	0.19
Total secondary	0.46	**0.02**
Ratio primary/secondary	− 0.40	**0.05**

Nonparametric correlations with Spearman rank coefficients (Rs). Other relationships to SeHCAT, including the percentage free, glyco-, tauro-, sulfo-conjugates, glyco/tauro ratio, CA/CDCA ratio were not significant.

Calculation of the predictive values gave the sensitivity and specificity of %PBA > 10% to detect a SeHCAT of < 15% of 45% and 63%. These were improved using a %PBA cut-off of 15% and SeHCAT < 10% (Table 6).

**Table 6:** Predictive values of % primary BAs for SeHCAT.

%PBA	SeHCAT (%)	Sensitivity (%)	Specificity (%)	PPV (%)	NPV (%)	Diagnostic odds ratio
> 10	< 15	45	63	63	45	1.39
> 15	< 15	45	88	83	54	5.83
> 15	< 10	56	90	83	69	11.25

%PBA, percentage primary bile acids; PPV, positive predictive value; NPV negative predictive value.

## Discussion

We have identified differences in bacterial diversity and metabolites, including SCFA, VOC and BA, between cohorts of patients with well-defined BAD and IBS-D. Overall reduced bacterial diversity was observed in BAD, but a greater abundance was found of certain anaerobic taxa, including specific members of *Lachnospiraceae, Bifidobacteria, Prevotella, Verrucomicrobia* and *Bacteroides*. It is unclear whether this results from the effects of the higher concentrations of BAs entering the colon or, if this is also a causative factor in the development of the disease.

Increasing, and sometimes conflictory, information relate the gut microbiome to the metabolome and BAs. For example, cholic acid feeding increased Firmicutes and reduced Bacteroidetes in rats^27^. However Bacteroidetes decreased less than Firmicutes in our BAD patients despite increased colonic BAs. The relative abundance of Bacteroidetes has been shown to correlate with the proportion of propionate^28^, and we found also increased propionate in BAD. Presumably, particular propionate-producing species become more abundant in BAD, and excess BAs selectively reduce other species^7^.

In addition to the effects of gut microbiota, SCFA production may also be altered in both BAD and IBS-D by gastrointestinal transit time, motility and physiology, and the amount and type of fermentable substrate ingested, especially if patients have already modified their diet to help with symptoms. The clear relationships we found of percentage faecal water with total SCFA, acetate, butyrate and propionate support this, and show that the effects of BAs, indicated by the SeHCAT, on most SCFA were small.

Looking more broadly at the metabolome, the separation of VOC profiles between the IBS-D and HC cohorts, and between the BAD and HC groups supports the notion that VOCs reflect the metabolic processes of the microbiota, with evidence of dysbiosis in both BAD and IBS-D cohorts. The differences in the branched SCFA levels contribute to this. Some, like isobutyrate were lower in the BAD compared to IBS-D and HCs, but others like isocaproic were higher. These result from protein fermentation, where amino acids are utilized by the colonic bacteria^29^. Differences in precursors supplied to the microbiota, with different enzymatic processes, produce variation in metabolic end-products, and in the VOC fermentation ‘chemical print’.

The dissimilar faecal BA composition of BAD and IBS-D cohorts is are not unexpected given the differences in bacterial diversity observed between the two groups. The increase in unconjugated, primary BAs in BAD in faeces and in serum is likely due to reduced biotransformation in the conversion of primary to secondary BAs^6^. There are multiple bacterial species, including many members of the Bacteroidetes phylum, that express forms of BSH and are capable of the first step of deconjugation^8,30^. Fewer species express enzymes for 7α-dehydroxylation to secondary BAs. These are predominantly Firmicutes, with various species of *Clostridium* (such as *C. scindens*) and the *Ruminococcus* family, (including former *C. leptum*)^6,8^. Reduction in their abundance in BAD will contribute to the increases in faecal primary BAs and ratio of primary/secondary BAs.

Faecal ursodeoxycholic acid was higher in BAD, which suggest that the several species (mostly *Clostridia*) with HSDH responsible for 7α/β epimerisation were maintained^6,8^. We have only limited species-level data to address this. An increase in faecal sulfated BAs was also observed in BAD. The liver is the predominant site of 3-sulfation, which decreases BA toxicity and enhances elimination. In the colon, various bacteria, including *Clostridia* species, can desulfate BA, allowing reabsorption^31^. The increase may be a further adaptive change to alleviate BA accumulation in BAD and a protective mechanism in intestinal dysbiosis^32^.

The concept of dysbiosis-driven changes in BA metabolism reflects data from earlier studies of unselected IBS-D patients. These demonstrated increases in primary BAs, decreases in secondary BAs and changes in microbiota, including increased *C. leptum*, and suggested that altered BA transformation by a changed microbiome was a driver for BA dysregulation in IBS-D^10,18^.

Two recently published studies have expanded these findings significantly^19,20^. In a large and thorough study, Zhao et al*.* identified a subset (24%) of IBS-D patients with higher total BA on a single stool sample (> 90th-centile of controls) and compared them to control and IBS-D subjects with lower BA^19^. Their cut-off was 10.61 µmol∙g^−1^ stool, slightly higher than our median of 9.17 µmol g^−1^. These BA + IBS-D patients had not undergone SeHCAT testing, but had fasting C4 and FGF19 values similar to other cohorts with BAD^4^. As in our current study, faecal primary BA (CDCA and CA) and UDCA were higher in their BAD group. Faecal bacterial α-diversity did not differ between their two groups although the β-diversity (instability) was higher in BAD. Their taxonomic analysis showed their BAD group had increased Firmicutes, and an abundance of Clostridia, including *Ruminococcus* species and *C. scindens*. These were then related to BA production (C4), feedback (FGF19) and to BA-transforming enzymes (BSH and HSDH). It should be noted that their group definition was BA in a single stool sample, rather from 7-day SeHCAT test. Potentially, adaptation of the microbiota and BA synthesis may occur with more prolonged BA loss.

Faecal microbiomes and metabolomes in IBS patients, including a subset with SeHCAT tests, were investigated by Jeffery et al*.*^20^ Principal component analysis of the microbiota was able to separate those with severe BAD (SeHCAT < 5%) from those with less severe BAD or normal SeHCAT. Analysis of the metabolome could separate groups with BAD, identifying glycerophospholipids and oligopeptides among the main predictive metabolites, although BA were less clear.

We have shown that the faecal % primary BA is inversely related to SeHCAT. This extends studies from the Mayo clinic, which suggested this measurement detects a group of BAD patients with different characteristics to those with elevated total BA^12,33^. (Their diagnostic cut-off for % PBA is > 10% and total faecal BAs > 2337 µmol/48 h. With a median 516 g stool/48 h^12^ this is equivalent to > 4.5 µmol g^−1^, close to the median of our SeHCAT-negative IBS-D group. Their group median total BA of 6.67 µmol g^−1^ is also lower than our SeHCAT positive group). We used a single stool sample, which is more variable and affected by diet, but is more user friendly than a 48 h collection on a defined diet. The Mayo group have recently extended their findings to also use a single stool sample^33^. We found that predictive values improved, to a diagnostic odds ratio of 11, using 15% PBA to detect moderate BAD (SeHCAT < 10%). However, we demonstrate (Fig. 3) a wide variability in the BAD group, indicating there may be several different mechanisms involved. Whether other microbial or metabolomic findings can be similarly developed for diagnostic purposes is unclear.

Our study has several limitations. The 16S rRNA sequencing mostly analysed bacteria at the genus taxonomic level. Some genera abundant in both cohorts could differ if information on species was available. This would have improved microbial characterization, but as BA metabolism, VOC and SCFA production occur via overlapping functional groups, this assessment might not have provided further answers regarding overall functionality and pathogenesis. More information could have been obtained with larger numbers, but our findings help identify areas for further confirmatory studies.

In summary, our results show that BAD patients, defined by SeHCAT testing, exhibit intestinal dysbiosis, altered BA metabolism and SCFA production, with a distinct VOC profile. The percentage of primary BA, dependent on metabolic changes produced by multiple bacteria, can predict SeHCAT and detect patients with BAD. The functional output of the microbiota, rather than abundance of specific taxa, may thus be more important in producing changes in BAD.

## Materials and methods

### Study population

Patients were recruited as part of the FAMISHED (Food and Fermentation using Metagenomics in Health and Disease) study. Scientific and ethical approval was acquired from the local Research and Development Office as well as Warwickshire Ethical committee ref: 09/H1211/38. Written informed consent was obtained from all participants in the study. In line with good medical practice and research governance framework, the study was undertaken in accordance with relevant guidelines and regulations.

Patient and public involvement–This study was reviewed and presented at the Bile Acid Diarrhoea charity (https://www.bad-uk.org) meeting led by patients. The aims of the study were presented and outline especially of recruitment process was discussed. Specific advice was sought in that regard and changes made to improve recruitment pathway. The study received support from the group and upon publication, results will be disseminated on the charity website and Facebook patient group. Study results were also presented at the UK Bile Acid Diarrhoea Network annual meeting.

Participants with chronic diarrhoea were included in the BAD or IBS-D groups based on ^75^SeHCAT testing. BAD was diagnosed with a ^75^SeHCAT 7d retention value ≤ 15%; and categorised as severe if < 5%, moderate if between 5–10% and mild if between 11–15%. The diagnosis of IBS-D was based on Rome III criteria with a negative ^75^SeHCAT test. Patients with inflammatory bowel disease (IBD) or a previous cholecystectomy were only included if ^75^SeHCAT was < 15% (type 1 and type 3 BAD respectively). Participants were excluded if they suffered from coeliac disease, active IBD (faecal calprotectin > 50 μg/g or CRP > 11 mg/L), colorectal cancer, antibiotics/probiotics use in the last three months, or recent bile acid sequestrants. HCs had no evidence of chronic disease, were not on any regular medications, pregnant or had taken antibiotics/probiotics in at least three months. Urine, stool and serum samples were collected in standard specimen collection bottles at 9am and stored at − 80 °C within 2 h.

### Faecal gut microbiome analysis: 16S rRNA sequencing

Stool DNA was isolated from 200 mg of stool samples using the QIAamp Fast DNA stool extraction kit (QIAGEN, UK), following the manufacturer’s protocol for pathogen detection. Methods were similar to those previously published^34^. To amplify genes for coding and sequencing, polymerase chain reaction (PCR) was used. V3-V4 primers and extensor ready mix (Thermo scientific) were used to amplify the 16S rRNA gene V3-V4 gene fragment from isolated metagenomic DNA using a PCR thermal cycler program. After PCR, the DNA samples were separated and sequenced using the ILLUMINA MISEQ V2 2 × 300 bp paired end protocol.

Default Illumina software trimmed sequences to remove adapter sequences, primers, barcodes, low-quality sequences and those with < 1000 reads. Using a custom Java program, formation of contigs was performed by joining together forward and reverse reads with a quality filtering step to remove contigs that had more than three mismatches. The contigs were de-replicated and then clustered at 97% identity to form operational taxonomic units (OTUs) using the UPARSE pipeline. Singleton contigs from the dataset were discarded and chimeras were removed. The UPARSE pipeline calculated the abundance of each OTU by mapping the de-replicated contigs against the OTUs sequences. Taxonomy was assigned to 16S RNA gene OTU sequences using QIIME (Quantitative Insights into Microbial Ecology) and the RDP classifier. Using the QIIME pipeline, the level of alpha diversity in our samples was determined by generation of rarefied OTU tables, computing measures of alpha diversity for each rarefied OTU table, collating the rarefied OTU tables and then generating rarefaction curves. The depth of rarefaction was defined by either the lowest number or median number of sequences assigned to a sample within a group that was analysed. The Shannon index was used to calculate alpha diversity indexes from rarefied samples. Rarefaction was performed on OTU tables to remove sample heterogeneity.

### Faecal SCFAs

Absolute and relative quantification of SCFAs (C2-C8) and branched-chain fatty acids (isobutyrate, isocaproic and isovaleric) was undertaken using gas chromatography in diethyl ether extracts as described previously^35^. Moisture content (faecal water content %) was measured the day prior to SCFA analysis with the samples being freeze dried for 24 h in an Edwards apparatus (Freezer Dryer Micro Modulyo). Each sample was measured in duplicate to improve accuracy of results. Data are presented per mass of dry faecal material (µmol g^−1^) and as the proportion (%) of total SCFAs.

### Analysis of urinary VOCs

The samples were analysed using a commercial field asymmetric ion mobility spectrometry (FAIMS) instrument (LONESTAR, Owlstone, Cambridge, UK), fitted with an ATLAS headspace sampling system. This instrument operates at room temperature and pressure, undertaking separation of complex chemical mixtures by measuring ionised molecular movement in high-electric fields. By scanning through a range of electric field strengths and compensation voltages, the instrument is able to produce a mobility map of a sample^24^.

Frozen urine samples were defrosted; 5 ml was aliquoted into a 20 ml vial and placed in the ATLAS sampling system (no cap is fitted), set to 40 °C and left for 10 min to equilibrate and generate an VOC headspace. Clean synthetic air is the passed over the sample and into the Lonestar instrument. The instrument was set to sweep the dispersion field between 0 to 90% in 51 steps, the compensation voltage from − 6 to + 6 V in 512 steps and both positive and negative ion measurement undertaken, resulting in 52,254 data points being generated per sample^24^.

### Measurement of faecal and serum bile acids

Serum and faecal BA profiles were generated using high-performance liquid chromatography coupled to tandem mass spectrometry (HPLC-MS/MS). Faecal samples were prepared by the addition of ammonium carbonate to release the BA from the binding protein, followed by centrifugation and solid-phase extraction using reversed-phase silica Chromabond C18 cartridges (100 mg; MACHEREY-NAGEL, Duren, Germany) for pre-analysis clean up.

An analytical column (Pinnacle II C18, RESTEK, Lisses, France; 250 × 3.2 mm) with a 5 µm silica particle (RESTEK) fitted on an HPLC binary pump (AGILENT 1100; Agilent Technologies, Massy, France) was used for the chromatographic separation of BAs. Mass spectra were obtained using an API 2000 Q-Trap (AB-SCIEX, Concord, Ontario, Canada) equipped with a turbo ion-spray (ESI) source. Analyst software (version 1.4.2, AB-SCIEX) was used to acquire the data.

The total primary BAs is the sum of CA and CDCA and their respective glyco-, tauro- and sulfo-derivatives. The total secondary BAs is the sum of LCA and DCA and their respective glyco-, tauro- and sulfo-derivatives, as well as hyodeoxycholic acid and its tauro-derivate.

### Statistical analyses

Data for patient demographics are presented as means and standard deviations (SD). The data for SCFA and BA are presented as medians with interquartile ranges (IQR). VOC data were analysed using a previously developed processing pipeline^24^. Kruskal–Wallis analyses were used to compare 3 groups and the Mann–Whitney U test was used for two groups. Spearman rank correlations were used to identify associations. *P* values < 0.05 were considered statistically significant.

## Supplementary information

Supplementary Information.

## Data Availability

The datasets generated during and/or analysed during the current study are available from the corresponding author on reasonable request.
