# Supplementary material for: The pathophysiology of bile acid diarrhoea: differences in the colonic microbiome, metabolome and bile acids
Source: Sci Rep. 2020 Nov 24;10:20436. doi: 10.1038/s41598-020-77374-7 (PMC7686486; doi:10.1038/s41598-020-77374-7)
Supplement: Supplementary file 1 — Supplementary Information. [file 41598_2020_77374_MOESM1_ESM.docx]

# The pathophysiology of bile acid diarrhoea: differences in the colonic microbiome, metabolome and bile acids.

Nidhi M Sagar, Henri Duboc, Gemma L Kay, Mohammad T Alam, Alfian N Wicaksono, James A Covington, Christopher Quince, Margarita Kokkorou, Valos Svolos, Lola J Palmieri, Konstantinos Gerasimidis, Julian RF Walters, Ramesh P Arasaradnam

# Supplemental Tables

**Supplemental Table 1: Demographic data of the study participants**

|  | Study | BAD | IBS | Healthy controls |
| --- | --- | --- | --- | --- |
| **Number** | Microbiome | 20 | 14 | **--** |
|  | SCFA | 20 | 20 | 26 |
|  | VOC | 13 | 26 | 13 |
|  | sBA | 10 | 8 | **--** |
|  | fBA | 10 | 9 | **--** |
| **Age** | Microbiome | 56.9 ± 12.4 | 45.3 ± 15.6 | **--** |
| (years) | SCFA | 55.4 ± 13.1 | 55.4 ± 13.1 | 55.4 ± 13.1 |
| (Mean ± SD) | VOC | 47.8 ± 14.6 | 43.0 ± 13.6 | 34.5 ± 9.3 |
|  | sBA | 50.9 ± 19.3 | 45.3 ± 17.5 | **--** |
|  | fBA | 52.3 ± 19.0 | 45.1 ± 19.2 | **--** |
| **Gender** | Microbiome | 60:40 | 57:43 | -- |
| (% F:M) | SCFA | 85:15 | 85:15 | 58:42 |
|  | VOC | 46:54 | 81:19 | 61:39 |
|  | sBA | 50:50 | 87:13 | -- |
|  | fBA | 70:30 | 67:33 | -- |
| **Ethnicity** | Microbiome | 90:10:0:0 | 86:14:0:0 | -- |
| (% C:SA:AC:O) | SCFA | 90:10:0:0 | 95:5:0:0 | 96:0:4:0 |
|  | VOC | 92:8:0:0 | 89:8:4:0 | 62:23:15:0 |
|  | sBA | 100:0:0:0 | 100:0:0:0 | **--** |
|  | fBA | 100:0:0:0 | 89:11:0:0 | **--** |
| **BMI** | Microbiome | 28.7 ± 6.5 | 27.8 ± 5.6 | -- |
| (Mean ± SD) | SCFA | 28.5 ± 8.1 | 27.3 ± 5.1 | 22.4 ± 2.6 |
|  | VOC | 30.0 ± 7.2 | 28.5 ± 7.0 | **--** |
|  | sBA | 27.9 ± 4.9 | 26.9 ± 3.6 | **--** |
|  | fBA | 26.4 ± 4.6 | 30.0 ± 9.9 | -- |
| **SeHCAT %** | Microbiome | 7 ± 4 | 34 ± 20 | **--** |
| (Mean ± SD) | SCFA | 7 ± 4 | 39 ± 10 | -- |
|  | VOC | 5 ± 3 | 36 ± 10 | -- |
|  | sBA | 5 ± 3 | 33 ± 21 | -- |
|  | fBA | 7 ± 3 | 27 ± 15 | -- |
| **Severity of BAD** | Microbiome | 15:40:45 | NA | -- |
| (% Mild:Mod:Sev) | SCFA | 20:30:50 | NA | -- |
|  | VOC | 7:31:62 | NA | -- |
|  | sBA | 0:40:60 | NA | -- |
|  | fBA | 10:60:30 | NA | -- |
| **Type of BAD** | Microbiome | 15:70:15 | NA | -- |
| (% T1, T2, T3) | SCFA | 10:70:20 | NA | -- |
|  | VOC | 8:69:23 | NA | -- |
|  | sBA | 10:70:20 | NA | -- |
|  | fBA | 20:70:10 | NA | -- |

Numbers of subjects and demographic and clinical data for the various studies are shown. SCFA= short chain fatty acids, VOC=volatile organic compounds, sBA=serum bile acids, fBA=faecal bile acids. Ethnicity is shown as C=Caucasian, SA=South Asian, AC=African Caribbean, O=other. BMI= body mass index. Severity of BAD is the percentage categorised as mild, moderate (Mod) or severe (Sev). T1, T2, T3 are types 1, 2 and 3 BAD (see text.) Data are means and SD.

**Supplemental Table 2: Abundance of OTU by significance of increase in BAD vs. IBS-D**

| **OTU** | **Chi**^2^ | ***p*-value** | **Mean BAD** | **Mean IBS-D** |
| --- | --- | --- | --- | --- |
| OTU_136 | 10.28 | 0.0013 | 1.98e-02 | 4.05e-03 |
| OTU_283 | 10.28 | 0.0013 | 6.59e-04 | 4.55e-05 |
| OTU_17 | 9.39 | 0.0022 | 7.39e-02 | 1.25e-05 |
| OTU_268 | 8.82 | 0.0030 | 2.05e-04 | 0 |
| OTU_519 | 8.54 | 0.0035 | 2.38e-04 | 5.38e-06 |
| OTU_72 | 8.27 | 0.0040 | 1.50e-02 | 8.20e-04 |
| OTU_127 | 8.00 | 0.0047 | 1.62e-02 | 0 |
| OTU_356 | 8.00 | 0.0047 | 3.64e-04 | 5.38e-06 |
| OTU_319 | 7.22 | 0.0072 | 1.87e-04 | 0 |
| OTU_553 | 6.97 | 0.0083 | 7.95e-05 | 0 |

The 10 OTUs with the highest Chi^2^ comparing abundance in BAD and IBS-D are shown, with uncorrected *p* values and the mean proportions in each group.

The OTU were assigned to the following taxa:

OTU_136 p__Firmicutes; c__Clostridia; o__Clostridiales; f__Lachnospiraceae; g__; s__

OTU_283 p__Actinobacteria; c__Actinobacteria; o__Bifidobacteriales; f__Bifidobacteriaceae; g__Bifidobacterium; s__longum

OTU_17 p__Bacteroidetes; c__Bacteroidia; o__Bacteroidales; f__Prevotellaceae; g__Prevotella; s__copri

OTU_268 p__Firmicutes; c__Clostridia; o__Clostridiales; f__Lachnospiraceae; g__; s__

OTU_519 p__Firmicutes; c__Clostridia; o__Clostridiales; f__Ruminococcaceae; g__Ruminococcus; s__

OTU_72 p__Bacteroidetes; c__Bacteroidia; o__Bacteroidales; f__Bacteroidaceae; g__Bacteroides; s__

OTU_127 p__Bacteroidetes; c__Bacteroidia; o__Bacteroidales; f__Prevotellaceae; g__Prevotella; s__copri

OTU_356 p__Firmicutes; c__Clostridia; o__Clostridiales; f__Ruminococcaceae; g__; s__

OTU_319 p__Verrucomicrobia; c__Verrucomicrobiae; o__Verrucomicrobiales; f__Verrucomicrobiaceae; g__Akkermansia; s__muciniphila

OTU_553 p__Bacteroidetes; c__Bacteroidia; o__Bacteroidales; f__Bacteroidaceae; g__Bacteroides; s__

**Supplemental Table 3: Associations of the proportion of individual SCFA with total SCFA in each group.**

| **Total SCFA** | | | | | | |
| --- | --- | --- | --- | --- | --- | --- |
|  | **Healthy controls** | | **IBS** | | **BAD** | |
|  | **Rs** | ***p*** | **Rs** | ***p*** | **Rs** | ***p*** |
|  |  |  |  |  |  |  |
| % Acetate | ‑ 0.39 | **0.03** | ‑ 0.17 | 0.24 | ‑ 0.45 | **0.02** |
| % Propionate | 0.16 | 0.22 | 0.43 | **0.03** | 0.37 | 0.06 |
| % Butyrate | 0.40 | **0.02** | 0.57 | **0.004** | 0.35 | 0.07 |
| % Isobutyrate | ‑ 0.55 | **0.002** | ‑ 0.60 | **0.003** | ‑ 0.38 | 0.05 |
| % Valeric | 0.24 | 0.12 | ‑ 0.44 | **0.03** | ‑ 0.06 | 0.40 |
| % Isovaleric | ‑ 0.58 | **0.001** | ‑ 0.64 | **0.001** | ‑ 0.64 | **0.001** |
| % Caproic | 0.25 | 0.11 | ‑ 0.30 | 0.10 | ‑ 0.85 | **0.001** |
| % Isocaproic | ‑ 0.11 | 0.29 | ‑ 0.52 | **0.009** | ‑ 0.45 | **0.02** |
| % Heptanoic | 0.19 | 0.17 | ‑ 0.13 | 0.29 | ‑ 0.63 | **0.001** |
| % Octanoic | 0.09 | 0.34 | 0.07 | 0.39 | ‑ 0.59 | **0.003** |
|  |  |  |  |  |  |  |

Data from healthy controls (n=20), IBS‑ D (n=18) and BAD (n=20) groups.

Rs = Spearman rank correlation coefficients.

P values <0.05 are shown in bold.

**Supplemental Table 4: Faecal bile acid proportions in patients with IBS-D or Bile Acid Diarrhoea**

| **Percentage of total faecal BA** | **IBS-D** | | |  | **BAD** | | |  | ***P* values** |
| --- | --- | --- | --- | --- | --- | --- | --- | --- | --- |
|  | **Median** | **IQR** | |  | **Median** | **IQR** | |  |  |
|  | | |  |  |  |  |  |  |  |
| Primary BA | 7.2 | (3.1–11.6) | |  | 14.3 | (3.8–64.2) | |  | 0.41 |
| CA | 3.1 | (1.7–4.9) | |  | 9.0 | (1.8–47.6) | |  | 0.33 |
| CDCA | 4.5 | (1.5–7.8) | |  | 6.9 | (1.7–15.7) | |  | 0.33 |
| Secondary BA | 92.5 | (87.6–95.9) | |  | 79.1 | (25.0–95.6) | |  | 0.22 |
| DCA | 66.8 | (54.3–77.3) | |  | 58.9 | (21.1–71.7) | |  | 0.22 |
| LCA | 20.3 | (13.9–29.3) | |  | 15.7 | (3.5–25.6) | |  | 0.12 |
| UDCA | 0.7 | (0.2–1.1) | |  | 2.0 | (0.2–9.0) | |  | 0.28 |
| Glycoconjugates | 1.2 | (0.9–4.4) | |  | 0.9 | (0.4–1.5) | |  | 0.14 |
| Tauroconjugates | 0.6 | (0.4–1.0) | |  | 0.5 | (0.1–0.8) | |  | 0.37 |
| Sulfoconjugates | 0.7 | (0.4–1.8) | |  | 0.8 | (0.6–10.0) | |  | 0.46 |
| Unconjugated BA - Urso | 95.4 | (92.4–97.1) | |  | 90.8 | (75.7–98.3) | |  | 0.46 |
| Ratio primary / Secondary | 0.1 | (0.0–0.1) | |  | 0.2 | (0.0–5.3) | |  | 0.37 |
|  |  |  | |  |  |  | |  |  |

The median and IQR are given for the percentage of total faecal BA from IBS-D (SeHCAT >15%; n=9) and BAD (SeHCAT <15%; n=10).

Comparisons were made by Mann-Whitney U-Tests.
